# Analysis of implementation processes in a hybrid effectiveness-implementation trial of interpersonal psychotherapy (IPT) for major depressive disorder in prisons: Training, supervision, and recommendations

**DOI:** 10.1371/journal.pone.0288182

**Published:** 2024-05-14

**Authors:** Jennifer E. Johnson, Maji Hailemariam, Caron Zlotnick, Fallon Richie, Shannon Wiltsey-Stirman

**Affiliations:** 1 Charles Stewart Mott Department of Public Health, College of Human Medicine, Michigan State University, Flint, Michigan, United States of America; 2 Department of Psychiatry and Human Behavior, Warren Alpert Medical School, Brown University, Providence, Rhode Island, United States of America; 3 Butler Hospital, Providence, Rhode Island, United States of America; 4 University of Cape Town, Cape Town, South Africa; 5 University of North Carolina at Charlotte, Charlotte, North Carolina, United States of America; 6 Dissemination and Training Division, National Center for PTSD, Menlo Park, California, United States of America; 7 Department of Psychiatry and Behavioral Sciences, School of Medicine, Stanford University, Palo Alto, California, United States of America; University of Nicosia, NEPAL

## Abstract

**Background:**

There are 10 million admissions to U.S. prisons and jails each year. More than half of those admitted have mental health problems. The goal of this article is to inform: (1) implementation of evidence-based mental health treatments in prisons and jails, an important effort that needs more evidence to guide it; (2) psychotherapy and interpersonal psychotherapy (IPT) training efforts, especially in low-resource settings.

**Methods:**

A randomized hybrid effectiveness-implementation trial of group IPT for major depressive disorder (MDD) in state prisons found that IPT increased rates of MDD remission and lowered posttraumatic stress disorder symptoms relative to prison treatment as usual. The trial used prison counselors, only some of whom had prior psychotherapy training/experience, to deliver IPT. IPT treatment adherence was high (96%), but trial training and supervision were too costly to be scalable outside the trial. The current article reports results from a planned qualitative analysis of 460 structured implementation and supervision documents in that trial to describe training and supervision processes and lessons learned, inform training recommendations, and facilitate future work to optimize training and supervision for under-resourced settings.

**Results:**

Themes identified in implementation and supervision process notes reflected: work on psychotherapy basics (reflective listening, focusing on emotions, open-ended questions, specific experiences), IPT case conceptualization (forming a conceptualization, what is and is not therapeutic work, structure and limit setting, structure vs. flexibility), IPT techniques (enhancing social support, role plays, communication analysis), psychotherapy processes (alliance repair, managing group processes), and managing difficult situations (avoidance, specific clients, challenging work settings). Counselors were receptive to feedback; some relied on study supervisors for support in managing stressful prison working conditions.

**Conclusions:**

Findings can be used to make future training and supervision more efficient. Based on our results, we recommend that initial and refresher training focus on IPT case conceptualization, steps for addressing each IPT problem area, and reflective listening. We also recommend supervision through at least counselors’ first two rounds of groups. More low-cost, scalable training methods are needed to get mental health treatment to individuals who need it most, who are often served in challenging, low-resource settings such as prisons. This is a mental health access and equity issue.

**Trial registration:**

The trial was registered at clinicaltrials.gov (NCT01685294).

## Introduction

There are more 10 million admissions to U.S. prisons and jails each year [1, 2]. More than half of those admitted have mental health problems [3]. Major depressive disorder (MDD) is the most common serious mental illness among individuals who are incarcerated [3, 4]. A national survey of state prisoners found that 23.5% met criteria for MDD within the past year, three times the national past-year prevalence [3]. In-prison consequences of MDD can include dropout from correctional treatment programs, inability to assertively protect oneself, physical victimization, aggressive acting out, and increased suicide risk [5–9]. Once out of prison, MDD increases risk of recidivism [10]. Therefore, it is important to know how to implement evidence-based MDD treatments within prisons, potentially unique and challenging implementation climates [11, 12].

Unfortunately, research guiding implementation of evidence-based mental health interventions in justice settings is sparse. More is needed. This article describes implementation processes in a randomized hybrid effectiveness-implementation trial [13] of Interpersonal Psychotherapy (IPT) for MDD in two state prison systems [11, 14]. Results of that trial, the first fully-powered (n = 181) randomized MDD treatment trial in any incarcerated population, indicated that IPT reduced depressive symptoms, hopelessness, and posttraumatic stress disorder symptoms, and increased rates of MDD remission relative to prison treatment as usual [14]. As a result of the trial [14], which evaluated the effectiveness and cost-effectiveness of IPT for MDD in prisons, IPT became the only MDD treatment for incarcerated individuals supported by evidence from a full-scale randomized trial.

IPT was chosen because of its strong evidence-base in non-incarcerated populations [15, 16], its fit for the target population, and pilot data suggesting that it is acceptable and effective for MDD among incarcerated women [17, 18]. IPT’s focus on addressing recent stressors, such as interpersonal conflicts, life changes, and grief, is a good fit for incarcerated populations, who are faced with many of these disruptive life events [19].

Cost is key for mental health implementation in prisons because prison systems can often afford few licensed mental health providers [11]. Task-shifting may increase access to care. Previous studies in this line of research have demonstrated that non-specialist bachelor’s-level prison counselors can conduct IPT [20]. The trial included both master’s-level mental health professionals and non-mental health specialists (such as bachelor’s-level re-entry planners) working in the prisons as study IPT counselors [11].

The ultimate goal of this line of work [11, 14] was to determine whether psychotherapy in prison could be done in a way (i.e., group, non-specialist counselors) that is effective and cost-effective enough to be offered in prisons regularly [11, 14]. Trial results suggest that the answer is “yes,” if training and supervision costs can be decreased without losing effectiveness. Supervision in this trial was effective in that IPT produced better outcomes than did prison treatment usual, and prison counselors learning IPT for the trial (some of whom had no prior formal psychotherapy training) were highly adherent (spending an average of 96% of time in sessions on model) and reasonably competent (averaging 5.1 on a 1 to 7 scale) [14]. However, 72% of the costs of IPT in the trial were for training and supervision (including study supervisor and counselor hours) [14]. Given extremely sparse prison mental health training budgets, this is not scalable or sustainable. On the other hand, single workshops typically have little effect on provider competence [21]. Results of the current analysis can help optimize future training and supervision to make it more efficient for wider-scale implementation.

This article reports results from a planned qualitative analysis of 460 structured implementation and supervision documents in this randomized effectiveness-implementation trial [14] to describe training and supervision processes and lessons learned. As noted above, effectiveness and cost-effectiveness results from the trial have been published [11, 14]. Johnson et al. [22] reported the first part of implementation analyses, addressing the first four Consolidated Framework for Implementation Research (CFIR [23]) domains that describe how characteristics of the intervention, the inner setting (i.e., implementing facilities), outer setting (i.e., policies), and individuals involved (i.e., counselors, clients) affect implementation. The current report is the third planned paper in the series, analyzing items that had been top-level coded for the fifth and final CFIR dimension, implementation processes.

This article describes two sets of analyzes applied to items top-coded within the CFIR process dimension. The first analysis summarizes implementation processes using thematic analysis of activities within the CFIR process steps (planning/engaging and *executing*/reflecting/evaluating). Detailed implementation/training steps are rarely published and present a contribution to the psychotherapy training literature. The second analysis identifies common themes in discussions between supervisors and study counselors. The first analysis (description of implementation/training steps) provides context for the second (common themes in what went well and what was a challenge in supervision in this trial). Taken together, this information can guide decisions to use similar processes or adapt them in the future.

Results from these analyses can inform and optimize: (1) implementation of evidence-based mental health treatments in prisons and jails, an important effort that needs more evidence to guide it; (2) psychotherapy and interpersonal psychotherapy (IPT) training efforts generally, especially in low-resource settings. Analysis of training and supervision processes and lessons learned in the first major MDD trial among incarcerated individuals can inform psychotherapy training recommendations and optimization for prisons and other low-resource settings with high mental health need. Efficient training in such settings is important to mental health access and equity. To our knowledge, this is the first detailed description and analysis of implementation processes of a mental health intervention in a prison or jail setting.

## Methods

The hybrid effectiveness-implementation trial randomized 181 individuals with MDD who were in prison to IPT (n = 91) or to prison treatment as usual (n = 90) [11, 14, 22]. As described above, results favored IPT across multiple outcomes, IPT treatment adherence was high (96% of time on model), and IPT treatment competence was acceptable (average of 5.1 on a 1 to 7 scale) [14]. The current manuscript reports prospectively collected implementation data from prison-based IPT counselors and IPT supervisors in the trial, as they worked with the 91 clients randomized to the IPT condition. IPT training and supervision took place from November 2011 to December 2014.

### Ethics approval and consent to participate

This trial was approved by Brown University’s Institutional Review Board (FWA 00004460) and regulatory bodies overseeing prison research in participating states. All study procedures were carried out in accordance with relevant guidelines and regulations. Study investigators had access to counselor and patient participant identities during the study. Written informed consent was obtained from participants before beginning study procedures.

### Sources of data

Data for this analysis consisted of a systematic, planned document review of 100% of the existing documentation kept by the study team throughout the study period (2012–2014), in order to create a structured description of implementation as it occurred. Prison counselors provided IPT for the study at 3 women’s facilities and in 3 men’s medium security facilities in two northeastern U.S. states as part of the randomized trial [14]. These counselors were trained and supervised in IPT by study clinical supervisors (JJ and JN). Document review data sources included structured process notes of clinical supervision sessions (n = 359) kept by study clinical supervisors, relevant email exchanges between the study and prison staff (n = 46), minutes from study team and prison meetings (n = 18), internal memos (n = 10), and other files that documented implementation processes (official letters, training manuals and intervention goals; n = 27) [11, 14, 22]. To aid with the planned document review, structured supervision process notes kept by the study team included the following questions: “What IPT elements went well?”, “What challenges were encountered? What was recommended?” “How did the counselor respond to the feedback?” “Specific barriers/facilitators discussed at the facility, counselor, client, or state prison system level.” [11, 14, 22].

### Interventions

#### Treatment as usual

Prison treatment as usual for MDD typically consists of antidepressant medications (either selective serotonin reuptake inhibitors or tricyclics) [24]. Psychosocial interventions in prison are often psychoeducational and highly structured. IPT was not being provided in participating facilities other than through our study.

#### Group IPT

Group IPT used the study treatment manual [11, 25]. IPT was offered to 91 study participants who had MDD, were incarcerated at participating prisons, and were randomized to the IPT condition in the trial [14]. It was delivered using 20 90-minute group therapy sessions over 10 weeks with 4 individual (pre-group, mid-group, post-group, and maintenance) sessions. Individual sessions focused on treatment planning, individualized treatment goals, and preparing clients to use group effectively. In the pre-group individual session, counselors reviewed what depression is and some of its causes, explored potential precipitating events for the current depressive episode, explained how those problems are typically addressed in IPT, and then worked with participants to choose target problems and IPT approaches for their situation (i.e., create a mutually agreed upon treatment contract). As in Wilfley et al. [26] we asked counselors to summarize the case conceptualization (which we called “goals summaries”) for clients’ review and revision at the first group session. Goals summaries included a simply worded description of the client’s precipitating crisis for MDD, its link to the current depressive episode, and IPT steps for addressing it. Because the prisons are mental health resource-poor, counselors almost always ran groups alone rather than in pairs. The mean and median number of members per group was 4; the largest group had 7 members [14]. Members were assigned to IPT groups based on logistical considerations (when they enrolled in the study and the facility in which they were housed, such as men’s medium security, women’s facility, etc.).

#### Training and supervision

The study hired prison counselors to offer IPT as moonlighters. Counselors were employed at participating prisons, with at least a bachelor’s degree and at least one year of prison work experience. Study clinical supervisors were external to the prisons. Study supervisors provided study counselors an initial 1.5-day IPT training (with all study counselors together) and then ongoing individual IPT supervision. Three refresher trainings were also offered during the 3-year study (see details in the results section). Ongoing supervision consisted of weekly review of counselors’ audiotaped IPT sessions, feedback on their written goals summaries for each client, and weekly individual phone consultation. When the secondary supervisor was supervising a counselor, the primary supervisor provided supervision of supervision. This 3-year, real-world pragmatic effectiveness trial did not have counselors practice and become certified in IPT before beginning groups. After the initial training, counselors began groups under study supervision. Therefore, notes made on the training and supervision process cover counselors’ entire IPT learning trajectories. This approach is also closer to what is realistic in in low-resource settings.

### Qualitative data analysis

The approach to analysis used thematic analysis following the steps of framework analysis [27]. The Consolidated Framework for Implementation Research (CFIR) [23] was used as the *a priori* framework for the larger series of papers related to the trial. Johnson et al. [22] reported findings from the CFIR constructs of inner setting, outer setting, intervention, and individuals involved. The current report analyzes items that had been top-level coded for the fifth and final CFIR dimension, implementation processes.

Within the CFIR process domain, codes were analyzed two ways. First, we briefly summarized implementation processes using thematic analysis of activities within the CFIR process steps (planning/engaging, and executing/reflecting/evaluating [23]). Second, we used thematic analysis to identify themes in discussions between supervisors and study counselors within each section of the structured process and supervision notes. Supervision was guided by three prompts for supervisors to discuss with counselors: (1) What went well? (2) Where do you feel like you struggled? (3) Tell me about your case conceptualization for each client. Supervision notes (made by supervisors after supervision) followed a semi-structured template that included supervisors’ and counselors’ perspectives on what went well in the session/s, challenges the counselors experienced, and recommendations offered by the supervisor/receptiveness of the counselor to the supervisor’s recommendations. We used thematic analysis to identify themes within responses to each of these sections (i.e., what went well, challenges, and recommendations/response sections).

Prior to coding, JJ and SWS created a comprehensive codebook for the study. The team coded the documents using NVivo software. Data was coded by four study team members (MH, FR, JJ, and a 4^th^ coder) with previous qualitative experience and familiarity with the study topic. JJ was also the study principal investigator and one of the study supervisors. The four coders met as a team to establish consensus after coding the first 10 files independently. The coders coded the rest of the data individually, meeting in pairs to establish consensus. When discrepancies emerged, coder pairs discussed and reached agreement; final consensus files were integrated into the master file for analysis. Within responses top-coded within the CFIR “processes category,” we first briefly summarized implementation processes within the CFIR process steps (planning/engaging, and executing/reflecting/evaluating [23]). Second, thematic analysis was applied first to responses to the “what went well?” section of the process notes, then to responses to the “challenges” section of the process notes, and then to responses to the “recommendations/counselor response to feedback” section of the process notes, yielding three sets of themes. MH was the primary qualitative analyst with secondary interpretation and validation by JJ.

In the results section, supervisors are labeled “S01” and “S02,” and counselors are labeled “C01” through “C11.” Clients receiving group IPT are referred to as “group members” (i.e., members of the IPT treatment groups).

## Results

A total of 460 supervision notes, meeting minutes and email exchanges between the 11 counselors and the two supervisors describing implementation and supervision were analyzed. Results are organized by analyses. The first set of analyses describes implementation processes pulled from the 460 study documents categorized and organized using the CFIR process steps. The second set of analyses identified themes within each section of the structured supervision notes: (a) counselor and supervisor perspectives on what went well, (b) counselor and supervisor perspectives on challenges, and (c) supervisor recommendations and counselor receptiveness.

Both study supervisors were white, non-Hispanic women in their 30s. Nine of the 11 study counselors were female. Eight counselors were non-Hispanic white; the others were Hispanic White (n = 1), Asian (n = 1), and Non-Hispanic Other (n = 1). Counselors had a median age of 33 (range 26–49) at the end of the 3-year trial. Individuals enrolled in the trial (n = 181) were 35% female and 19% Hispanic, with self-identified race described as White (59%), African American/Black (20%), Native American (2%), Asian (1%), more than one race (6%) and Other (12%). Their average age at study enrollment was 39 (range 20–61) [14].

### Analysis 1: Implementation processes categorized using CFIR [23] process steps

Analysis of implementation documentation using the CFIR process steps yielded the following summary of implementation processes. It is important to understand these processes to contextualize what went well and what was a challenge in subsequent supervision. Together, this information can guide decisions to use similar processes or adapt them in the future.

#### Engaging and planning

Many prison specialist and non-specialist counselors indicated interest in being study counselors. Counselors described receiving additional training, supervision, and support, as well as getting paid by the study to work outside their extra hours, as motivators. Our initial training cohort included 7 prison counselors (4 non-specialists and 3 masters-level social workers), 5 of whom ended up leading IPT groups (one was in a small facility that did not have enough participants and another became overcommitted). We hired and trained an additional 4 counselors (all master’s prepared prison mental health clinicians) over the course of the study to cover new facilities or existing facilities as study counselors got promoted. We trained most of these new counselors at refresher trainings or abbreviated versions of the original training. We had to train one new counselor (C06) in only two 2-hour segments due to a last-minute schedule switch, and she required additional training over the course of her first group.

Initial training of study counselors took place over a day and a half, on a Friday evening and a Saturday to work around counselors’ prison workweek. An outline of training topics and methods is shown in Table 1. Training procedures accommodating both licensed mental health counselors and non-specialist counselors had been honed in our pilot studies of IPT in prisons [17, 18] and followed our prison IPT manual [11, 20]. Based on training experiences in the pilot trials, training for this trial (1) emphasized that the manual should not be read word for word when delivering IPT; (2) used audio recordings of IPT to give counselors practice responding and a sense of what IPT sounds like; and (3) offered recorded mock sessions covering more complicated IPT techniques, like communication analysis, that counselors could borrow and watch. Counselors said the most helpful aspects of this training were role plays, videos, and the lunch we provided. As a result of feedback on the first training from counselors and supervisors, we added additional detail on the pre-group IPT session, finding a current (rather than past) interpersonal goal, provided additional example recordings, and planned refresher trainings to help counselors learn from each other’s experiences.

**Table 1:** Initial training as provided.

Topic	Time	Details
**Friday Evening Session (4 hours)**		
Overview	15 min	Welcome, introductions, and overview
MDD	20 min	Definition, rates, treatment options
IPT rationale	30 min	Social support, IPT focus areas, and their relationship to MDD: grief, interpersonal conflicts, life changes, problem patterns.
Counseling essentials	70 min	The client is the expert on his/her own experience, needs, desires, future (why; role play to have trainees experience what it is like to be on the receiving end of direct/teach vs. listen/evoke helping styles) Empathy and reflective listening (what it is, what it is not, small group role plays to give everyone a chance to practice and to be on the receiving end, responding to audio recordings as a group) Emotions (don’t fix, just listen) The power of ounsellor expectancies for clients
IPT case conceptualization, pre-group session	45 min	Introduction and education about MDD Practice interpersonal circle exercise Role play practice identifying the interpersonal problem area How to create written interpersonal goals summaries
Role play entire pre-group session	50 min	Group leaders demonstrate and let trainees watch Trainees can lead small parts as they feel comfortable
Discussion and homework	10 min	Homework: practice reflective listening with friends in at least 3 conversations, record at least 1 (we provided audio recorders) Finish reading the treatment manual, if not already finished
**Saturday Session (8 hours)**		
Welcome	15 min	Welcome and questions
Counseling essentials	45 min	Discussion of reflective listening homework (listen to trainee audio recordings as a group: where do you think you did well? Where do you feel like you struggled? Pointers)
Study logistics	45 min	Case notes, audio recorders, confidentiality, emergencies
IPT techniques	75 min	Discussion, lived and taped demonstration, role plays of: ounsellor as client advocate, empathy, validation, building a therapeutic alliance, non-judgmental approach, working with ambivalence, clarification, communication analysis [with role plays], problem-solving, helping clients role play, present relationship focus, helping group members (i.e., clients) support each other, attention to group process, strategies that are never part of IPT
Grief sessions	45 min	Big picture and details of addressing grief in IPT Role play reflective listening in grief work
Lunch and walk	30 min	We provided lunch
Conflicts sessions	75 min	Big picture and details of addressing interpersonal conflicts in IPT Watch a videotaped demonstration Role play communication analysis Role play helping clients change unhelpful communication patterns
Life changes sessions	30 min	Big picture, details, and role play
Problem patterns sessions	45 min	Big picture and details Role play reflecting listening in problem patterns work Role play working with ambivalence in problem patterns work
Strategies for challenging situations	45 min	Using the group, managing group process (e.g., monopolizers, quiet people, etc.), suicidality, conflict or hostility in the group, outside conflicts spill over into the group, trauma or dissociation, guardedness, violence or threats in relationships
Synthesis and summary	30 min	Review of big picture and important points, adherence and competence measures, IPT quizzes, question and answers Reflective listening Help clients resolve interpersonal problems, including accepting mixed feelings about grief, conflicts, life changes, problem patterns Work to create a respectful, supportive relationship with clients Emotions matter; help clients experience and express them. Help clients develop social support. If you are stuck, “What is that like for you?” and “Who else can you discuss this with?” are almost always good questions
Feedback	15 min	Final questions. Feedback on the training and the manual.

Note: Training followed the structure of the intervention manual. We sent the manual to counselors several weeks before the training and asked them to read the manual and come with questions.

#### Executing, reflecting, and evaluating

Training continued with ongoing supervisor audio recording review and weekly individual telephone supervision. In particular, supervisors listened to counselors’ pre-group IPT sessions and helped them to edit and refine the written IPT case conceptualizations (“goals summaries”). Supervisors also observed that both specialist and non-specialist counselors seemed to understand IPT much better after their first set of groups.

*After having run [IPT] groups a few times*, *[C02] said it was easier to run groups this time*. *She indicated that she had a better understanding of what the therapy was about and what needed to be worked on*. *She stated that the improved understanding of IPT made it possible for her to know where she was going so she could “think less” and focus on what she needed to do*. **-*S01 note on C02 supervision***

We conducted 3 refresher trainings during the 3-year study (at 6, 15, and 21 months after the initial training; Table 2) that covered areas in which counselors were still struggling. Our first refresher training reviewed the steps to forming a mutually agreed-upon written case conceptualization and negotiating a treatment contract before the beginning of the group. Counselors had struggled with these ideas, so we refined the pre-group IPT session in the manual and provided additional training in these skills. Negotiating the case conceptualization and treatment contract was the hardest part of IPT to learn, but once counselors learned it, they were well on their way to achieving IPT competence. The refresher training also covered focusing versus flexibility in IPT groups. The second refresher training reviewed IPT treatment details. A final (full day) training covered all previous trainings in a condensed fashion for an additional cohort of counselors and as review for ongoing counselors. Because these final counselors were all master’s trained mental health providers (i.e., specialist counselors), we trained them in 8 hours rather than 12. Clinical training took place in person. Ongoing clinical supervision took place individually by telephone.

**Table 2:** Refresher trainings as provided.

Topic	Details
**Refresher training 1 (half day; 6 months after initial training)**	
IPT case conceptualization	It is important to have a mutually agreed-upon written case conceptualization/ treatment contract (“goals summary”) before the beginning of the group. The goals summary is written for the client in lay terms. It provides a brief description of the problem leading to the current depressive episode and ways of using the IPT group to address it.Steps for negotiating the treatment contract/goals summary include: 1. Determine when the client’s *most recent* (not first) depressive episode started (or their mood worsened, if client has chronic MDD) 2. Ask open-ended questions about what was going on in the client’s life around that time. Ask about their relationships, any losses, any changes in their circumstances. 3. Listen carefully for interpersonal conflicts, life changes, grief, and isolation. With individuals in prison, there are usually many potential triggers. 4. Reflect potential triggers for the depressive episode back to the client: “So, it seems like there were a lot of stressful things going on in your life at the time,” list them, then ask, “Which of them bothered you or is currently bothering you the most.” 5. Discuss the most salient triggers for the current depressive episode, and then once one (or two) are identified, ask, “Would you like to work on that issue in group”? 6. Once a troublesome issue that the client would like to work on is identified, describe the IPT steps for working with that issue, how s/he could use the group, and ask him/her if that makes sense. 7. Negotiate and renegotiate as necessary. 8. Then finally, write up a user-friendly paragraph describing the issue and another short paragraph describing how to address it. 9. Bring the statement to the first group, run it by the client, and revise as necessary. Interpersonal (i.e., therapeutic) goals may be refined over time, but it is important to start group with an interpersonal goal in mind. Reviewed written examples.
Common IPT errors	Get the triggers of the CURRENT depressive episode, not the first one. Interpersonal goals should be present focused. If a client identifies a trigger from the far past, ask “Are there things going on now in your life that remind you of that time?” Get a treatment contract (i.e., interpersonal goals that the client wants to work on in group) before group starts
Focus vs. flexibility	Reviewed steps to address each IPT problem area How to decide what to work on in a session How to balance emergent issues on any given day with what you had planned for IPT work that day, especially when counselors are still learning IPT
IPT essentials	Review and importance of reflective listening and empathy When in doubt, use reflective listening. This can be used to slow things down and re-establish an alliance if something feels adversarial or the client feels misunderstood
Discussion, reflection	What has gone well? What has been a challenge? Lessons learned from specific cases
**Refresher training 2 (half day; 15 months after initial training)**	
IPT finer points	Different ways of solving interpersonal conflicts (communicate, set boundaries, change expectations, end the relationship). The goal is to move clients from a stuck, ambivalent place, not necessarily to get them to communicate with someone they’ve given up on. Meeting clients where they are: respecting their reading level, readiness to discuss issues in group Appropriate level of detail to explore sensitive issues (e.g., trauma history, crime details) Importance of a structured mid-group individual session to review progress on goals and discuss work to do before the end of group Importance of a formal goodbye at the last session Ideas for handing challenging clients, especially Axis II, in group
IPT essentials	Review and importance of reflective listening and empathy
IPT case conceptualization	IPT case conceptualization is the purpose of the pre-group session. Review of steps. When did the mood change? Reflect the challenges you hear in an IPT frame and ask them what they think is most important (it is the client’s goal). Writing the goals summary.
IPT approach	The 2–3 steps for addressing each interpersonal problem area Practicing communication analysis
Discussion, reflection	Summaries of recent groups Wins, challenges, lessons learned.
**Refresher training 3 (full day; 21 months after initial training)**	
Overall	This was a 1-day training and refresher training condensing all the topics from previous trainings review for ongoing and learning for new study counselors

### Analysis 2: Themes observed within each section of process and supervision notes

Themes corresponding to each section of the structured supervision notes (i.e., what went well, challenges, and recommendations/responsiveness) are described below. Themes in each section are underlined. A summary of themes in each section is shown in Table 3.

**Table 3:** Supervision themes.

	What went well	Challenges	Recommendations and responsiveness
Therapy basics	1. Reflective listening and empathy 2. Focusing on emotions 3. Getting experience/conversation specifics 4. Open-ended questions	1. Reflective listening, focusing on emotions 2. Getting experience/conversation specifics	1. Reflection, emotions, specificity, open-ended questions
IPT case conceptualization	5. Case conceptualization, advancing work on interpersonal goals 6. Structure/limit-setting/protecting clients 7. Structure with flexibility	3. Pre-group session/treatment contract/case conceptualization 4. Counselors off-model 5. Keeping the group focused and doing IPT work 6. Managing structure vs. flexibility	2. Case conceptualization 3. What are the next IPT steps? 4. Keeping the group focused and doing IPT work 5. Limit-setting and structure 6. Structure vs. flexibility
IPT techniques	8. Role plays, communication analysis, work on interpersonal conflicts		7. Role plays, communication analysis, social support
Therapy processes	9. Alliance and alliance repair (including negotiating tasks, goals) 10. Group processes	7. Managing group process/conflicts	8. Managing group processes
Difficult situations		8. Challenging clients 9. Clients don’t want to feel/discuss feelings 10. Other forms of resistance (attendance, disinterest in social support) 11. Counselor schedules, frustrations with the prisons	9. Challenging clients
Counselor growth	11. Counselors putting the pieces together and getting more confident		10. Encouraging, supporting counselors 11. Counselor receptiveness

#### What went well

Themes found within the “what went well” section of supervision notes included: (1) reflective listening and empathy; (2) focusing on emotions; (3) getting specifics of client experiences and conversations; (4) asking open-ended questions; (5) case conceptualization and advancing work on clients’ interpersonal goals; (6) structure, limit-setting, and protecting clients; (7) structure with flexibility; (8) specific IPT techniques such as role plays, communication analysis, and work on interpersonal conflicts; (9) alliance and alliance repair (including negotiating tasks and goals of treatment); (10) group processes; and (11) counselors putting the pieces together and becoming more confident.

Supervision notes focused on several core IPT skills that are central to most psychotherapies (i.e., therapy basics). Reflective listening and empathy were among these core skills. Not all counselors had been trained to use these techniques prior to the study, but all used them well during the study, sometimes to great effect.

*[Client] started out with some [resistance] at first*, *but [C03] was careful to validate and empathize and [the client] really engaged and did some good work…*. *[The client] sounded angry*, *[C03*] *said*, *you sound angry*, *he said*, *I’m not angry*, *[another client] said*, *I don’t know what you’re feeling man*, *but you sound pretty [expletive] angry*, *and [the first client] started to cry*. ***-S01 note on C03 supervision***

Reflective listening helped counselors elicit clients’ emotions about situations being discussed and create the safety needed for clients to experience those emotions, another core skill of IPT. Several of the counselors had been trained in alternate therapy styles, which tended to ask about thoughts or behaviors or focus on providing education, rather than on experiencing emotions. However, all the counselors (including the non-specialist counselors) became good at listening for, focusing on, and helping group members (i.e., clients) talk about emotions over the course of supervision.

[*C02] asked a group member to share her story*. *[C02] attempted to maintain focus on slowing the participant down and getting the details of the complete narrative as well as eliciting the emotions that the participant felt at that time*. *[C02] consistently does a good job eliciting emotional content from the group members*. **-*S02 notes from C02 supervision***

Helping clients slow down and provide specifics about their experiences and conversations in their personal narratives is another core IPT skill. Supervision notes described several times when counselors did a good job inviting clients to describe the specifics of a grief or life change narrative (to elicit and process emotion) or of a conversation that did not go well (to role play and problem-solve how to communicate better in the future; “*Could you go through what you said line by line*?”). Open-ended questions also helped clients experience emotions. Supervision notes detailed examples of excellent questions, including questions focused on exploration (“*How does this conflict with your brother affect you*?”), process affect (“*What was it like having that conversation*,*” or “What is it like to talk about this now*?*”)*, and building social support *(“How can we support you*?*” and “When you’re struggling and you need help*, *who can you go to*?*”*).

One of the more difficult skills for counselors to master was keeping track of group members’ case conceptualizations and knowing how best to focus the group to advance work on interpersonal goals. Some counselors understood this well on their first set of groups, but most did better by their second set of groups.

[*C03] did a really nice job–making spot-on clinical judgments about what the core IPT issue is*, *what in-group issues to address and not*, *and doing some really nice reflecting and following the model*.–***S01 note on C03 supervision***

[*C11] has a good sense of the women in group*, *their interpersonal goals*, *and where they need to go in the remaining groups*.–***S02 note on C11 supervision***

Advancing work on interpersonal goals required clarity about the goals and methods used in group to achieve them, a clear agenda, structure, limit-setting, the ability to redirect less productive conversations, and the ability to protect group members. Supervision comments related to such structure included, “*Good review of confidentiality and expectations/logistics of group*” and “*Good group format*: *check-in*, *session topic*, *and check-out*.*”*

*Group was giving [the client] a lot of advice and “you should have known”*…. *Counselor defended [her and said it wasn’t her fault]*. ***-S01 note on C04 supervision***

[*C07] does nice job giving overview of conflict session and the kinds of information she is interested hearing about to understand the conflict*. ***-S02 note on C07 supervision***

When counselors understood the bigger picture arc of IPT treatment and the steps to address each category of interpersonal problem, they were better able to have a structure but adapt in real time if other relevant issues surfaced (i.e., structure with flexibility).

[*C11] has done a good job structuring groups*, *so the women know what the main focus of the group is*, *whose “turn” it is for the day*, *as well as rolling with things when they don’t go exactly as planned*. ***-S02 note on C11 supervision***

The next set of skills often commented on in supervision notes was specific IPT techniques, including communication analysis (a line-by-line telling of a conversation to determine how better to do it next time), role plays, and work on interpersonal conflicts. One example of successful communication homework included:

[***C07]*** *did a nice job giving a group member some concrete interpersonal homework (try not to say the “f-word” for two days and see if people stick around longer to talk to you*, *try to find some new guys in the program to help)… He laughed about the first assignment but agreed to both*, *and tried them and by Thursday*, *was happier*. *Said he’s definitely getting different reactions from others*. ***-S01 note on C07 supervision***

Supervision notes often commented on therapy processes such as therapeutic alliance (including alliance repair).

[*C01] has a nice way with the clients*. *She was client*, *understanding*, *and they seem to open up to her*. *It’s obvious that she wants to do a good job and that she is there to help*. ***S01 note on C01 supervision***

[*C06] did a nice job addressing the issues from last week’s groups; she was clear and direct with the group members about her not hearing what they were asking her for last week*. *The group members responded well*. -***S02 note on C06 supervision***

Therapeutic alliance also includes agreement on goals and tasks of treatment. Supervision notes sometimes commented on how the mutually negotiated treatment contract and other choices provided by the counselors helped group members take ownership of their own therapeutic work.

*How to give people options about their interpersonal goals and have them decide so they feel like they own them*, *they then take responsibility for them and it’s great. -**C07 as quoted in S01 notes.***

[*C02] did a nice job checking in with the group member to see if she felt comfortable talking about a topic (e*.*g*., *her crime) and respecting the group member’s boundary about this*. *She also checked back in with her at the end of group and got the group member to agree that it would be helpful to talk about this with her other group members as well as use the group to talk through what she wants to communicate to her family and get feedback*.–***S02 note on C02 supervision***

Supervision notes often described counselors’ efforts to foster helpful group processes, including getting group members talking to each other, asking about commonalities among group members, helping quiet members speak and monopolizers listen, and teaching group members how to use reflective listening and provide empathic feedback to help each other.

[*C04] sent an email about Conflicts Session 2*, *saying that it was some of the best*, *most authentic work he’s ever seen*, *with excellent feedback from group members to each other*, *and that they gave each other honest feedback in one of the most skilled ways he’s ever seen… he was excited for me to hear it*.–***S01 note on C04 supervision***

The final category of comments in the supervision notes about what went well related to when all the IPT skills were coming together and counselors were proficient in the model.

[*C01] is empathizing*, *working with feelings*, *building support*, *seems to get the model*, *working on communication and relationships… IPT has been a different way of working for [C01] (more focused on feelings*, *less on strict behavioral activation)*, *but she is really*, *really enjoying it… can’t wait to start next group*. -***S01 note on C01 supervision***

#### Challenges

Supervision notes also had a section describing challenges that counselors experienced on the way to learning and mastering IPT. Themes found within the “what went well” section of supervision notes included: (1) reflective listening and focusing on emotions; (2) getting specifics of client experiences and conversations; (3) using the pre-group session well to be able to obtain a treatment contract and formulate a case conceptualization during that session; (4) counselors off-model; (5) keeping groups focused and doing IPT work; (6) managing structure vs. flexibility; (7) managing group processes and conflicts; (8) challenging clients; (9) clients not wanting to feel or discuss feelings; (10) other forms of resistance (e.g., poor attendance or disinterest in social support); and (11) difficult counselor schedules and counselor frustrations with the prisons.

In terms of therapy basics, counselors began at differing stages of experience with reflective listening and comfort focusing on emotions. Furthermore, even experienced counselors are not 100% empathic all the time.

*She’s telling him what to do rather than empathizing*. ***-S01 note on C07 supervision***

*He’s interpreting*, *rather than reflecting*. *“Sounds like you’re romanticizing”… [C03]* “*confronted him”… instead of just asking “do you want to come back or just do individual sessions or be done*?*” -****S01 note on C03 supervision***

*It’s hard for the counselors without mental health training to know how to follow and push a little for the emotionally intense stuff and how to do real therapy without getting caught up in* “*I have to fix this*!*” or “What is the next question on the manual*?*” It’s a confidence/anxiety issue*.*”* ***-S01 note on C02 supervision***

Supervision notes also included reminders to focus on specifics of group members’ experiences and conversations (rather than generalities), and to focus on the present (rather than the past).

The second set of challenges reflected learning IPT case conceptualization, which counselors typically understood better on their second set of groups. Supervision notes included documentation of some of counselors’ struggles with case conceptualization and negotiating a mutually agreed upon treatment contract in the pre-group session, especially initially. This was the most challenging part of learning IPT for counselors.

*Overall, the pre-group [IPT] sessions were short (45 minutes vs. 75) and though the beginning of the sessions started well*, *[C07] had trouble getting information she needed about when major depressive episodes started and ended and so didn’t leave the sessions with ideas about what treatment goals should be or with a treatment plan negotiated with the participants. She said she had felt “frustrated” in the sessions and didn’t know how to write the goals up afterward.–**S01 note on C07 supervision***

[*C08] indicated that she did better with the more structured groups than with the less structured groups*. *She also indicated that she sometimes would feel stuck and not be sure of what direction to go in next*. ***-S02 note on C08 supervision***

As a result of these challenges, our refresher training focused much more on the steps needed to create an IPT case conceptualization and negotiate a treatment contract in the first session (see Table 2). We also expanded the description of the first session in the treatment manual. In addition to learning IPT case conceptualization and how to get it in the first session, non-specialist counselors had to learn about the general idea of a case conceptualization, and more experienced counselors had to work not to default to other models.

[*C01] said that she had to work to keep her inner behaviorist in check and focus on feelings rather than pleasant activities when one woman was so sad*. ***-S01 note on C01 supervision***

These challenges were addressed through supervision and additional training sessions, including reviewing the IPT adherence and competence scales with counselors.

Another set of challenges related to keeping groups focused and doing IPT work. It took practice to hold the big-picture arc of each client’s treatment goals (i.e., case conceptualization) in mind to help focus moment-to-moment interactions. Counselors also needed to know when to set firm limits, including when group members pushed boundaries, tried to help other group members in unhelpful ways, were distractible, or when groups were off topic:

*Another challenge was that one of the group members was very vocal*, *very loud*, *and would run [the counselor] and everyone else over and clearly had his own agenda*… *When he went off*, *another group member would follow along*. *So*, *it was really hard for the first few weeks for C06 to get them doing interpersonal/therapeutic work because… there wasn’t a good therapeutic contract in place*. ***-S01 note on C06 supervision***

We trained this counselor very quickly when another counselor had a scheduling conflict. She had not fully understood how to make a collaborative therapeutic contract in the pre-group session. Once a good treatment contract was in place with each member, the counselor was able to help this group of clients do better IPT work.

One of the places the counselors struggled the most but mastered over time was managing structure versus flexibility in the IPT groups:

[*It was] hard for the therapist to hold onto the IPT frame in this session*. *I’ve had that with 3 therapists now I’ve trained*, *who have great exploratory/general skills*, *so they do well out of the gate*, *but then struggle a bit to stick to the structure of IPT*, *look at the manual*, *and then do the more active parts of IPT once they get closer to the middle of the group… In this active phase of group*, *I keep telling all of 3 them to focus solving the interpersonal problems and stick closer to the manual*. ***-S01 note on C01 supervision***

It took time for counselors to master what is core (e.g., IPT steps for working on grief, life changes, interpersonal conflicts) and what is flexible (e.g., session order) and when to go with what they had planned (typically have a 15-minute check-in and then conduct planned session) versus what walks in the door (when someone is in crisis or is upset about something related to work on one of the group member’s interpersonal goals). A common mistake was letting group check-ins go too long (e.g., 45 minutes) and not using more of the group time for interpersonal work.

Counselors also needed to manage challenging group processes, including conflicts among group members as well as members who monopolized group, derailed group, or gave unhelpful responses to other members (giving advice, shutting down emotions).

*Managing the argument kept the counselor on her toes*, *but she handled it well*. *At one point*, *a deputy came in to check on them (they were getting loud*), *which also helped*. ***-S01 note on C01 supervision***

Most conflicts among group members provided in-group opportunities to practice addressing conflicts. However, in two cases, we split groups because of conflicts that pre-existed the group and provided a distraction rather than a learning opportunity.

Counselors also sought supervision over clients they found difficult or frustrating. Most of the group members had personality disorders (72% had antisocial personality disorder and 38% had borderline personality disorder; Johnson et al., (2019) and almost all had difficult life histories.

*Therapist feels she struggles with one member who tends to talk and talk*… *Just when she thinks she should cut him off because what he’s saying is irrelevant, he says something that makes it relevant*. *We talked about how to manage him.–**S01 note on C07 supervision***

The most common resistance to IPT work that counselors experienced in groups was implicit or explicit avoidance of emotion:

[*C07] keeps trying to focus them on feelings and specific things*, *and they keep trying to move into general philosophy*.–***S01 note on C07 supervision***

*One group member believes that talking about things doesn’t make people feel better*. *He resists [C02’s] attempts to get more details about his life change or to share how he felt in past or currently. [C02] sticks with emotions as way to get him to identify feeling words, but this group member struggles to provide much detail about his interpersonal goals or use feeling words*.–***S02 note on C02 supervision***

Counselors also occasionally experienced resistance in other areas (attendance, the need for social support, role plays). Counselors addressed these challenges through re-establishing treatment contracts (“Given that, would you rather work on something else?” or “What would you like to do?”) and/or finding ways to accommodate clients’ concerns while doing other IPT work.

The final category of challenges documented in supervision notes was counselors being burned out, overcommitted, or frustrated with their other clinical work, with prison administration, or with their own life circumstances. Their prison positions were genuinely demanding.

*I’m just getting home now and I wanted to let you know that I had to cancel group tonight because of a crisis at the prison*. *I was able to call all of [the group members’] units to let them know*. -***C06 email to S01***

*Are you free to talk right now? Call with [C06] did not go well, she feels too frustrated with the Department of Corrections and everything and wants to bail*… *I am super frustrated*. ***-S02 email to S01***

*The circumstances of this group have been very*, *very hard for [C06] and this supervision took place by phone on a Friday night when she was sick after being at a funeral all week*. -***S01 note on above email from S02***

*[C05] is too busy*, *has rescheduled supervision [a lot]*.*… Supervision has been hard because we do it while she’s driving and she doesn’t take notes*… *This is her 3*^*rd*^ *job and she commutes 2 hours each way to her main job*. -***S01 note on C05 supervision***

#### Supervisor recommendations and counselor receptiveness

The final section of supervision notes described recommendations that supervisors made to counselors and counselors’ receptiveness to those recommendations. Themes found within this section included: (1) reflective listening, focusing on emotions, specificity, and open-ended questions; (2) case conceptualization; (3) recommendations for the next IPT steps; (4) keeping groups focused and doing IPT work; (5) limit-setting and structure; (6) structure vs. flexibility; (7) role plays, communication analysis, and social support; (8) managing group processes; (9) challenging clients; (10) encouraging and supporting counselors; and (11) counselor receptiveness.

The first large category of recommendations provided to counselors during supervision addressed the basic IPT and general therapy skills of reflection, focusing on emotions, asking for open-ended questions, and helping clients be specific in describing events and conversations. More use of reflective listening generally, as well as instruction to empathize and reflect rather than interpret or argue, was common feedback discussed during supervision.

*We have given her consistent feedback to use reflection rather than say “I understand what you*’*re saying*,*” which has also helped a lot*, *and she’s starting to do that beautifully*.–***S01 note on C06 supervision***

Feedback to ask for details of interpersonal communication and events was especially common for counselors without formal therapy training.

*Get more confidence pushing for details, specifics, and feelings*. *Don’t let them go with general, vague statements. **-S01 note on C02 supervision***

Supervisors also provided frequent feedback to help counselors with IPT case conceptualization, especially with the process of eliciting the information needed for the case conceptualization and then developing treatment goals collaboratively with clients.

[*Be sure to get] a treatment contract*: *“Here’s what I hear*, *here are our options for working*, *what do you think*?*” Provide options and then get agreement*. ***-S01 note on C07 supervision***

The intervention manual contained an “IPT cheat sheet,” with one page summarizing IPT goal areas and basic steps for addressing each. In addition, supervisors reviewed case conceptualization and implications for the next IPT steps for each group member during each supervision session.

*Discussed with group leaders the directions of where to go with each group member and what sessions to conduct next week*. ***-S02 note on C06 supervision***

*We discussed* [*C06] validating that it is okay to have feelings*, *okay to feel sad about losses*, *etc*. *We also discussed having [the client] focus on positive action-focused coping since he is trying to not get anxious and frustrated when his sister does not contact him or follow-through on things*. *He also values the relationship and most likely has not clearly communicated his feelings or needs to his sister*. *We discussed encouraging him to think through what is best for him and what he wants from this relationship*. *It is clearly a relationship that is highly valued*, *but also one that causes him stress and triggers his anger*. ***-S02 note on C06 supervision***

Recommendations to counselors also addressed strategies for keeping the group focused and working, as well as limit-setting and structure as needed. For example, supervisors sometimes suggested redirecting unhelpful responses among group members. Recommendations included:

*Eight group sessions left–what does she want to be sure to get done therapeutically before group is over*? *Talked about how this is a short period of time*, *it will go quickly and how to use the mid-group sessions and remaining group sessions to identify which work needs to be done and do it*. ***-S01 note on C06 supervision***

*One group member tends to monopolize group time and emphasized importance of… keeping him to reasonable check-in time*. ***-S02 notes on C06 supervision***

*After [he*] *told his grief story*, *one group member said*, *“you can’t change it*, *get over it*.*” Suggested cutting this kind of thing off*, *doing some education about grief*, *supporting validating feelings*, *and helping the group do that also*. ***-S01 note on C07 supervision***

Another theme of supervision recommendations was structure versus flexibility, and how to know when to do each. For example:

*I asked the counselor to follow the prescribed sessions more (he often never gets to them in the session or only as a brief “topic” at the end*). *He said part of the problem is that he’s trying to be flexible to go with whatever focus areas are hot for the group members that day*, *but it’s often something different than what he has prepared*, *and he can’t read the manual and run group at the same time*, *so it’s been hard to follow the manual and be maximally responsive to members’ needs when he doesn’t know the manual well yet*. *I told him for now*, *to go into group with one or two sessions prepared*, *to let them do their check-in and then to go with the prepared sessions*. *He has let them go a lot and now I think he can be more structured with specific work to get done*. ***-S01 note on C04 supervision***

*We talked about how to tell if someone is done with the emotional part of grief work (the feelings have subsided some*, *are less conflicted*, *and you know all the details of the story) and how to continue to get details of the story and help the woman have space to sort out her feelings about them*. *I also suggested not to try to “fix it” by talking her out of her guilt*, *but to let the woman talk it through and come to her own conclusions*. *Later*, *I had a very concrete suggestion for a woman*, *and [C02*] *said*, *“But isn’t that fixing it*?*” which was a great question*. *So*, *we talked about when a concrete suggestion makes sense (for a very concrete information-based issue*, *like “how do I get to the store*?*” or “you might try methadone treatment”) and when emotion-focused work makes more sense (an issue with a lot of conflicted emotions or that is very emotionally laden)*. *This made sense to her*.–***S01 note on C02 supervision***

Another common theme of supervisors’ recommendations included communication analysis, role plays, and helping group members build social support.

*Completed role plays… to improve communication since group member does not want to end relationships*. *Group leaders had group members start (so not to make a big deal about roleplaying)*, *but group members had trouble at first and would have benefited from a little more direction at the beginning*. *Provided feedback that one of the group leaders could start playing the group while the group member plays the other person (his wife in this example) since he has best idea of how wife might respond in the situation*. *Once the group leaders jumped in and completed the 2*^*nd*^ *role play*, *it went a lot better*, *the guys got a sense of how to do it and both liked this aspect of the session*. *Also discussed… suggestions to make to group member about his communication style*. *-****S02 note on C01 supervision***

Supervisors also frequently provided feedback or suggestions about making group processes and interactions among group members maximally useful.

*One group member shared a current stressor*… *One group member in particular tends to provide very directive feedback that can come across as unsupportive*. *I provided feedback to [C02] about how she could have slowed down this portion of group when the women were all talking at once and made sure that the group member who was sharing her story felt understood and to check in with her about how helpful the “directive” feedback is from the other group member*. -***S02 note on C02 supervision***

As mentioned above, a majority of clients in the study had personality disorder/s in addition to major depressive disorder. Therefore, some feedback to counselors related to helping counselors work with clients they found challenging.

*We discussed that… the main focus should be on the remaining work on her interpersonal goals*. *However*, *since these [personality] issues interfere with her social support*, *[C11] could also take opportunities to help her work on boundaries/finding healthy ways to get her needs met*. *This more positive frame might be something she could hear and be willing to work on and [C11*] *can more gently reflect both the things she does well and the things she struggles with around relationships*. ***-S02 note on C11 supervision***

Finally, as counselors were learning a new model (and sometimes learning psychotherapy itself as an entirely new skill), they tended to be hard on themselves. Much of the feedback supervisors provided was to encourage, support, and reassure the counselors.

*[C07*] *wasn’t sure about this session*, *if the guys are discussing what they need to*, *and feels that she has a hard time keeping one guy on track*. *I said I thought the session sounded great and the reasons why*. *She said she is still struggling to know when she’s on model or not and I told her the reasons she was on model and that she was doing great*. *She said until this session*, *the guys haven’t been talking to each other much and the guy who talked today has been quiet*. *I said*, *today was great*, *and they were even expressing appreciation for the things they learned from each other*! ***-S01 note on C07 supervision***

Counselor receptiveness. Prison counselors generally responded well to and appreciated supervision. There were only a few instances in which counselors were slightly defensive or subdued.

*They’re so happy they’re getting paid for supervision*, *because people pay a lot for supervision and don’t get good supervision like this*. ***-S01 note on C02 supervision***

*Regarding listening to the tape*. *I did*! *And I totally heard what you were referring to*. *I’m definitely planning on being more vigilant in my efforts to be reflective when interacting with* [*the client]*. ***-C03 email to S01***

In fact, there were several instances of counselors calling or emailing supervisors outside of their regularly scheduled meeting times to ask for help.

*I think I would like to talk to you before the next session*. *Today’s session did not go well both in and out of group*. *A few members got into an argument afterwards when talking to me and I would like to talk about ways to manage the next group*. *FYI*, *[client’s] father died today*, *which also added to the distress*. *I uploaded the session already*, *but there were many things not on the recording that need to be addressed*. *Do you still have Tues at 11*:*00 available to talk*? -***C04 email to S01***

## Discussion

### Current findings

This manuscript reports results from a planned qualitative analysis of 460 structured implementation and supervision documents in a hybrid effectiveness-implementation trial of group IPT for major depressive disorder in 6 state prisons. The goal was to inform: (1) implementation of evidence-based mental health treatments in prisons and jails, an important effort that needs more evidence to guide it; (2) psychotherapy and interpersonal psychotherapy (IPT) training efforts generally, especially in low-resource settings. To our knowledge, this is the first detailed description and analysis of implementation processes of a mental health intervention in a prison or jail setting. Analysis of training and supervision processes and lessons learned from the first major MDD trial among individuals who are incarcerated can inform and help to optimize psychotherapy training recommendations for prisons and other low-resource settings. Some study counselors had prior psychotherapy training and experience and some did not. Limitations of the study include having only two study supervisors and eleven study counselors. Study strengths included prospective, structured data collection documenting implementation processes, a rigorous qualitative coding process, and a unique and important implementation context (prisons) in which there has not been any previous analysis of counselor training processes.

The current qualitative analysis of supervision processes in this trial provides an outline for prison IPT training, retraining, and supervision (Table 1; Table 2). Themes identified in analysis of supervision notes (summarized in Table 3) also provide a detailed description of where training procedures produced hoped-for results and where additional retraining/focused attention was needed. Findings (Table 3) indicated that supervision focused on: (1) work on psychotherapy basics (reflective listening, focusing on emotions, open-ended questions, specific experiences); (2) IPT case conceptualization (forming a conceptualization, what is and is not therapeutic work, structure and limit setting, structure vs. flexibility); (3) IPT techniques (enhancing social support, role plays, communication analysis); (4) psychotherapy processes (alliance repair, managing group processes); and (5) managing difficult situations (avoidance, specific clients, challenging work settings). The single most challenging task to train was to help counselors understand the steps needed to formulate an initial IPT case conceptualization and negotiate a treatment contract in the pregroup individual session. This is a critical task because it frames everything else that is done in IPT, and a clear sense of the direction of therapy is needed to manage IPT in group settings, especially with frequent comorbid personality disorders (72% antisocial personality disorder and 38% borderline personality disorder in this trial [14]). Our initial training on obtaining an initial case conceptualization and treatment contract in the pregroup session had not been detailed enough (Table 1), so we revised our training procedures and focused on this IPT task at the refresher training (Table 2). We recommend focusing on this important task at length in future IPT trainings, especially in prisons. Counselors were receptive to feedback overall; some relied on study supervisors for support in managing stressful prison working conditions.

Based on the current analysis of supervision processes, together with previous findings related to treatment fidelity and outcomes in the trial [14, 22], we make several observations. First, supervisors’ observations in this analysis as well as previously published treatment fidelity and outcome results suggest that non-mental health trained providers can learn IPT [14, 22] using the training processes described in the current analysis. Second, all providers needed ongoing training in IPT case conceptualization to strike an appropriate balance between structure and flexibility in pursuing IPT goals in a group setting. A clear understanding of the big-picture steps of IPT allowed them better mastery of what is core and what is flexible. For those without previous psychotherapy training, it was important to explain the overall concept of case conceptualization. This included how it is possible to learn to keep a bigger picture of where the client headed in one’s mind while carrying on an in-the-moment conversation. We reassured new counselors that it gets easier with practice. Building in more IPT case conceptualization simulation and practice writing goals summaries during initial training may be helpful in reducing the learning curve for all counselors when groups begin. Our training had some role play of the initial session (see Table 1), but having counselors conduct and record a few mock first sessions and write the goals summaries might help. Third, group treatment was effective and cost-effective in this trial and in general [14, 28]. It is often necessary in settings with many clients and few resources. However, learning to track and manage multiple members’ therapeutic work in a group setting took time, especially for novice therapists. In other words, group is doable and likely necessary, but requires additional training support. Some of the counselors’ more challenging tasks (including the need to get a written treatment contract during a single pre-group session and managing multiple challenging group members) related to the group setting. Additional training in group skills and group processes [29] might be provided; however, the key challenge of the group setting was tracking multiple case conceptualizations simultaneously, which could be addressed by more training in the big picture steps of IPT. Fourth, given that a majority of group members had antisocial and/or borderline personality disorders in addition to MDD, it is remarkable that counselors had challenges with so few of them. This is a credit to the study counselors and may suggest generalizability/flexibility of IPT.

In the current analysis, study supervisors observed that both specialist and non-specialist study counselors seemed to understand IPT much better after their first sets of groups. This observation is consistent with previous findings that treatment effects of IPT in this trial (relative to treatment as usual) were driven by outcomes of counselors’ second and subsequent (not first) rounds of groups [14]. Therefore, training and regular supervision through at least the first 2 sets of groups seems ideal. In addition, counselors in this study needed and benefited from refresher trainings every 6–9 months over the 2 years of the trial (Table 1; Table 2). However, if training and supervision taper after that (and fidelity stays high), they become more scalable and sustainable. For example, previous cost analyses for the trial determined that costs for prison IPT programs that are already up and running (once ongoing supervision is no longer required) dropped from $2,054 per client to $575 [14]. However, even these scaled back procedures are still prohibitively expensive for most systems. Our hope is that by sharing a detailed analysis of the training process, we can facilitate future work to optimize training and supervision processes for under-resourced settings.

### Integration of current findings with previous findings from this trial

Current findings, in addition to previously published results from this trial provide reason for optimism for implementing IPT in prison settings. Effectiveness results [14] indicated that IPT reduced depressive symptoms, hopelessness, and posttraumatic stress disorder symptoms, and increased rates of MDD remission relative to prison treatment as usual alone. Previous analyses of the first four CFIR domains (intervention, inner setting, outer setting, individuals involved) outlined facilitators of IPT implementation in prisons. These included IPT being a good fit for the target population, counselors and prison group members being enthusiastic about IPT, and counselors being open to learning evidence-based practices and committed to helping their clients [22]. In fact, prison counselors, administrators, and group members were highly motivated to find ways to better address mental health problems in resource-poor prison settings [22]. Their dissatisfaction with what they could do with such limited prison resources and high perceived need to change something to better address client needs [22] was echoed in our current finding that counselors were highly receptive to feedback. This was an unusually motivated and open set of counselors to train.

However, limited prison mental health resources and high need at the center of implementation barriers observed in our previous analyses [22] were echoed in the current process analysis. The main challenges to IPT implementation identified previously were overcommitment of prison treatment staff and variable prison supervision and collegial support [22], due to prison financial limitations that limited the number of mental health staff hired and resources to support them. Findings in the current analysis underscored this point. As noted in the current analysis, prison counselors often relied on study supervisors for support in dealing with difficult working environments with limited supervision and collegial support. Study counselors leaned on study supervisors for the collegial support and guidance that was missing from their settings. High client needs (severity, comorbidity, high levels of trauma and life stressors) and challenging climates among correctional staff in some of the facilities exacerbated these challenges. Furthermore, this trial took place in two states that were in the lowest quintile of incarceration rates and among the better funded states, suggesting that conditions are likely worse elsewhere. This is concerning given that supervision is important for providing quality care and preventing burnout, compassion fatigue, and turnover [30, 31].

A critical IPT implementation task for the future is to find scalable training and supervision models to make them feasible for resource-challenged prison systems. In prisons and other low-resource settings (such as low- and middle-income counties) where supervisors are scarce and training time is limited, helping counselors become competent in IPT (and other evidence-based mental health interventions) as quickly and efficiently as possible is key for scale-up. In the current trial, supervision was effective in that IPT produced better outcomes than did prison treatment usual, and prison counselors learning IPT for the trial (some of whom had no prior formal psychotherapy training) had high IPT treatment adherence and acceptable IPT treatment competence [14]. However, 72% of the costs of IPT in this trial were for training and supervision [14]. Given extremely sparse prison mental health training budgets, this is not sustainable and scalable. On the other hand, single workshops typically have little effect on provider competence [21]. Lessons learned from the current analysis can inform and help to optimize training procedures that are both effective and more efficient for wider-scale implementation.

### Integration of current findings with findings from other low-resource settings

The need for more scalable approaches to psychotherapy training and supervision extend beyond prisons to other high-stress, low-resource settings such as refugee settlements, low- and middle-income countries, or World Health Organization Mental Health Gap Action Programs (mhGAP [32]). Similar to the U.S. prison context, implementation studies in these settings also highlight a lack of trained and qualified mental health professions and resource constraints as key barriers for scaling evidence-based mental health practices [33, 34]. Both our prison work and global mental health efforts have used remote supervision and task-shifting to lay counselors [35]. Our remote supervision was offered individually; other efforts [35–39] have successfully used remote group supervision. Giving counselors online access to example sessions [35] and recordings of the original training [38] for review can save some trainer/supervisor time. One project [37] asks counselors watch pre-recorded training segments prior to the virtual training. In the virtual training, after group review and questions, the counselors independently rate fidelity of pre-recorded “better” and “worse” sessions and discuss their answers with each other and the trainer. This helps to demonstrate interactions that do and do not fit within the psychotherapy model. Recording, automating, and using technology for training and supervision to the extent possible can help [40]. Many models now post freely available training and example videos online. Finally, train-the-trainer approaches or self-organized learning collaboratives can help to scale expertise. For example, experienced lay mental health counselors can play an important role in supervising other lay mental health counselors [41] and peer-to-peer supervision can be a sustainable quality assurance method [35]. However, all or almost all of these approaches have relied on outside (often research) funds to provide training, supervision, and technical assistance. Additional capacity-building approaches are needed.

## Conclusions and recommendations

We offer several recommendations for training professional and paraprofessional counselors in IPT. Based on current and previous [14, 22] findings from this trial, we recommend an initial training and at least one refresher training 6 months later that focus on: (1) the steps of IPT case conceptualization as outlined in Table 2 (how to involve clients in treatment planning while still guiding treatment; understanding which components should be counselor contributions and which should be client contributions to the plan), (2) how the steps of addressing each problem area (e.g., tell the story, feel the feelings, explore what is gained and what is lost, hold the memory and find new supports in the present) guide when to change gears or stay on course in a planned session, and (3) psychotherapy basics (reflective listening, open-ended questions, getting specifics of experiences and communication). We recommend providing supervision for at least the first two rounds of groups, including review of written case conceptualizations and at least some audio recordings. However, future efforts to streamline these processes are needed.

Finally, on a larger scale, incarcerating fewer people with mental illness and providing money in prison mental healthcare contracts for more counselors and more ongoing training and supervision would help address the resource shortages that make mental healthcare in prison settings so challenging [11], as demonstrated in this and previous [14, 22] analyses. More low-cost, scalable methods of training and supervision are needed to get mental health treatment to individuals who need it most, including those who are incarcerated and other mental health equity populations [14, 22].

## List of abbreviations and terms

C01: Counselor number 1. Counselors are labeled “C01” through “C11.”
CFIR: Consolidated Framework for Implementation Research
Group members.: Clients who were members of IPT study treatment groups
IPT: Interpersonal psychotherapy
MDD: Major depressive disorder
S01: Supervisor number 1. Supervisors are labeled “S01” and “S02.”
Study counselor.: Prison counselors who moonlighted for the study
